# User Preferences for Privacy Protection Methods in Mobile Health Apps: A Mixed-Methods Study

**DOI:** 10.5195/ijt.2020.6319

**Published:** 2020-12-08

**Authors:** Leming Zhou, Bambang Parmanto

**Affiliations:** 1 Department of Health Information Management, University of Pittsburgh, Pittsburgh, Pennsylvania, USA

**Keywords:** Mobile apps, Privacy, User experience

## Abstract

**Background:**

Mobile health (mHealth) apps have the potential to facilitate convenient health care delivery and self-management of health. However, many users have concerns about their privacy when they use mHealth apps. Different apps provide different solutions for protecting users' privacy.

**Objective:**

The purpose of this study was to determine user preferences among the several privacy protection methods used in current mHealth apps and the reasons behind their preferences.

**Methods:**

Five privacy protection methods currently used in mHealth apps were presented to a group of study participants who had mild or moderate depression and expressed concerns about privacy of information when they used mental health apps. After a demonstration of the methods, study participants were asked to fill out a questionnaire and indicate their perceived privacy protection level (PPPL) of each method, their preference rating for each method, and the privacy protection methods they had used in the past. A brief interview was then conducted to collect study participants' comments on these methods and elicit the reasons for their preference ratings. Statistical analysis was performed to determine the statistical significance of differences in participants' preference ratings and in the PPPLs obtained for the five methods. Study participants' comments on the privacy protection methods and suggestions were noted and summarized.

**Results:**

Forty (40) study participants were selected from a large candidate pool using the IRB approved selection criteria. All study participants viewed the app demonstration and understood the five privacy protection methods properly, which was indicated by their correct sorting of the PPPL of the five methods in their answers to the questionnaire. All study participants specified their preferences with respect to these methods and provided the rationale behind their selections on the questionnaire and during the brief interview. The results indicate that the users preferred privacy protection methods with *customizable modules in multi-purpose apps* because of their convenience and strong privacy protection, where the customization can be done either in the app or via a Web portal.

**Conclusions:**

This study identified user preferred privacy protection methods. These identified privacy protection methods may be used in many types of apps that perform sensitive health information management to better protect users' privacy and encourage more users to adopt these mHealth apps.

## BACKGROUND

Privacy is the freedom from unauthorized intrusion (Zhou et al., 2019). The unauthorized intrusion may occur *locally* or *remotely*. Remote privacy violations often occur via communication and information systems such as the internet after data breaches or unintended disclosure of personal information (Rendina & Mustanski, 2018). There are various approaches for preventing remote privacy violations, such as user education (e.g., never post sensitive personal information on the internet) and instituting security measures such as user authentication and data encryption (Kazatzopoulos et al., 2009; Maglogiannis et al. 2009; Martinez-Perez et al., 2015; Morera et al., 2016; Shafique et al., 2017; Smith et al., 2017). Local privacy violations are committed by people around a person; these are accomplished in various ways, including asking private questions, using personal items without authorization, viewing the person's internet browsing history on his or her personal computer or mobile device, and observing a person during private activities such as using a mobile health (mHealth) app (Boyles et al., 2012).

While providing security protection to personal health data is possible by using some security measures in mHealth apps, it is challenging to prevent local privacy violations in this field. One reason is because many mHealth apps have names including the target disorder, for instance, AIDSinfo, HIV & Pregnancy, Depression CBT, and PTSD Coach. Therefore, it is very easy for people around the app user to determine the nature of the app simply based on the app name alone. Moreover, while some mHealth apps may have more generic names, for instance, Talking Points, Life Check, Mood Tracker, and Mood Log, these apps are designed for one *single purpose* or disease. Therefore, a quick search of such app names on the internet reveals the nature and purpose of the app.

In both cases, the name of the app can trigger a local privacy violation even though others may not know whether the person uses the app or what contents have been entered into the app. This local privacy violation is based on the assumption that it is unlikely for people without any of these problems to have installed these apps on their phone, as indicated in one comment from a study participant in a previous study (Zhang et al., 2019):

“People will think only HIV infected individuals use the app, and my friend may doubt if I am HIV infected and may keep away from me.”

In other words, the existence of these apps on a person's mobile device can trigger a local privacy violation, even if other people may not see the users' private data, which may be well protected by various types of security measures.

Because of such local privacy violations, many users have expressed privacy concerns when they use mHealth apps to manage their own health despite the existence of various security measures within mHealth apps themselves (Atienza et al., 2015; Goldenberg et al., 2014; Kao & Liebovitz, 2017; Kenny et al., 2016; Xu et al., 2012). Security and privacy concerns about mHealth apps are greater when the apps are for issues associated with stigma, social isolation, or discrimination such as HIV (human immunodeficiency virus) infection, sexual orientation, and mental disorders (Atienza et al., 2015; Di Matteo et al., 2018; Goldenberg et al., 2014; Goldenberg et al., 2015; Kenny et al., 2016; Proudfoot et al., 2010). Therefore, although mHealth apps can be very helpful for making health assessment and therapy more accessible, efficient, and portable (Kao & Liebovitz, 2017; Nussbaum et al., 2019), if mHealth apps violate the privacy of app users, some users may choose not to use these apps (Atienza et al., 2015).

Specific to mental disorders, people with *mild or moderate* conditions may not have very obvious demonstrated symptoms but may mainly be experiencing a change in internal feelings such as an increase in hopelessness, sadness, or guilt (Beck et al., 1988; Kroenke et al., 2001; Radloff, 1977). Other people around those with mild or moderate mental disorders may not be able to easily tell whether these people have mental disorders. Similarly, people may have HIV infection but without any identifying symptoms (e.g., ones significantly different from a typical viral infection) for years (Feinberg & Keeshin, 2017).

Among those who have a stigma-associated disease but no obvious symptoms, different people make different choices in terms of *sharing* their own health information. Some choose to share their health problem with the people around them, such as family members, friends, classmates, and co-workers (Atienza et al., 2015; Zhou et al., 2019). Some choose to deal with their condition themselves and to keep their condition private. The latter group of people may consider using mHealth apps to assess and manage their condition (Crookston et al., 2017; Reid et al., 2013; Schueller et al., 2018; Switsers et al., 2018). However, they face a difficult choice related to the local privacy violation mentioned earlier. That is, they do not want other people around them to know that they have a silent health problem, but just having mHealth apps for this health problem on their mobile devices may reveal that information.

To protect their privacy, these people typically have two choices: either to not use mHealth apps to manage the health problem even though they may benefit from them, or to devise their own way to protect their local privacy (e.g., installing the app only when needed, or creating folders and hiding the app in those folders). These options may not provide the protection strength they desire and might introduce unnecessary inconvenience. A strong but convenient privacy protection method *embedded* in mHealth apps so that people may use these apps without any privacy concerns would be useful for these people.

## PREVIOUS WORK

In previous studies where researchers collected opinions about mHealth apps from people with mental disorders or HIV infection (Goedel et al., 2017; Goldenberg et al., 2014, 2015; Lipschitz et al., 2019; Proudfoot et al., 2010), results indicated that many desired the ability to obtain more information for their health needs via mHealth apps but had serious concerns about their privacy. Participants further revealed that due to these privacy concerns, they were hesitant to use mHealth apps for their health information seeking or health care self-management. The following briefly summarizes these previous studies.

Proudfoot et al. conducted an online survey (n=525), focus group discussions (n=47), and interviews (n=20) to investigate community attitudes towards adopting a mobile phone for mental health monitoring and management. The results indicated that people with depression, anxiety, or stress showed significantly stronger interest in this type of monitoring and management (p<.001), but they also expressed concern about intrusiveness and lack of privacy because of the monitoring activity. The authors mainly reported local privacy concerns from study participants and indicated some possible solutions, such as requiring user authentication, and providing alterable contents so that the monitoring could be used on public transportation (Proudfoot et al., 2010).

Goldenberg et al. (2014, 2015) performed focus group studies to determine the preferences of Men who have Sex with Men (MSM) for a mobile HIV app. Many of the study participants expressed interest in this app; however, they also expressed local and remote privacy concerns. In one study (Goldenberg et al., 2014), there were 38 MSM in the focus group discussions. In the other study (Goldenberg et al., 2015), there were 9 MSM in two phases of focus groups. Specific to local privacy concerns, study participants suggested that the app developers be “careful about icons and language, so that if others were to gain access to an app user's phone, they would not identify what the app is” (Goldenberg et al., 2014). The following are some quotations from the participants in the study, which clearly indicated their local privacy concerns: “*I could imagine if someone gets an HIV positive result, they're not going to want that to be something that oh, my little sister picks up my phone and sees this. So I would just be very thoughtful about how you designed those features…I think that would be critical to make sure that that's done in a way that minimizes the risk of any type of exposure that people don't want*.” “*I've had friends outed on various social media and apps and so even just having the icon of Grindr on someone's phone, it's a very distinct tell…I can only imagine if I wasn't out that would be something that I would be very concerned [about]. I don't know if I would keep an app like that on my phone at all, just because I wouldn't want to be found*.” “*I think the wording of [push notifications] would be pretty important not to have anything about HIV testing or something pop up on your screen. Your phone could be wherever*.” “*I am oftentimes in meetings and it's often me who's projecting up on a giant computer. The last thing I want is the schedule plus alert saying that it's time for me to get an AIDS test*.”

Goedel et al. (2017) performed a web-based survey to determine MSM's willingness to use mHealth apps for HIV prevention. In total, 169 MSM in London responded the survey. More than 60% (108/169, 63.9%) reported willingness to use a mHealth app for HIV testing reminders. The authors also mentioned desired features in terms of local privacy protection.

Lipschitz et al. (2019) did a cross-sectional survey study to investigate patients' interest in and barriers to adoption of mHealth apps for depression and anxiety. In total, 149 patients returned the survey. Most (87/149, 73.1%) reported interest in using an app for mental illness, but only 16 (10.7%) had done so. One of the most frequent concerns related to using an app for mental illness was data privacy (88/149, 59.1%).

Krebs and Duncan (2015) conducted a cross-sectional survey of mobile phone users throughout the United States. The results indicated that 427 study participants downloaded and used mobile health apps but stopped using some of these apps. Among these study participants, 29.0% (124/427) “did not like mobile health apps shared their data with friends,” which is a concern about local privacy.

Zhang et al. (2019) performed qualitative semi-structured interviews with 19 young MSM in China to determine their preferences for an HIV prevention mobile phone app. In this study, privacy was frequently mentioned, and the participants' concern was about local privacy with suggestions being related to the name and logo of the app. They did not want to have a gay-identified or HIV-identified app name or logo since that name or logo may cause “unintentional disclosure of the user's sexual orientation, or mistaken by others that the user is HIV infected” (Zhang et al., 2019).

It is worth noting that all the app users' privacy concerns are legitimate. In recent years, a large number of security breaches and privacy violations have been mentioned on the daily news and on government websites (Office for Civil Rights-US Department of State, 2019). However, many current mHealth apps do not provide sufficient protection of app users' data. For example, Grundy et al. (2019) performed traffic, content, and network analysis to investigate the data sharing practices of 24 selected mHealth apps. The results indicated that 19 of the 24 apps (79%) shared user data with various entities such as app developers, the vendor of the app, service providers, cloud service providers, and companies performing data analytics. The users of these apps may be aware of some of this sharing, but it is unlikely they are aware of all of them.

To encourage people with a strong interest in mHealth apps to actually use them, that is, to overcome their concern about data privacy, both remotely and local, one approach is to include user preferred privacy protection methods in mHealth apps. In this work, we specifically focus on gaining information about user preferences with regard to methods for preventing local privacy violations.

## OBJECTIVES

The aim of this study was to determine mHealth app users' preferred local privacy protection methods and the reason for their preferences. The results can guide mHealth app developers to create apps with desired privacy protection, which may then motivate more people to adopt mHealth apps for their own health management, especially those who are currently experiencing the dilemma of wanting to use mHealth apps because without them, they miss the opportunity of obtaining desired information for their own health, yet at the same time not wanting to use them because of concerns about local privacy associated with mHealth apps.

## METHODS

### STUDY PROCEDURE

To identify the app users' preferred local privacy protection method, we needed to use mHealth apps that handle data with a certain level of sensitivity. After all, if there are no sensitive data in the apps, there is no need for privacy protection. In this study, the sensitive data are the mental health information in mHealth apps. We recruited a group of people with mild or moderate depression to use these mental health related apps and express their preferences for local privacy protection methods in the apps. The results, however, may be applied to apps related to other type of health problems, such as HIV infection. The following is a brief step-by-step description of the entire study procedure

#### STEP 1: IDENTIFICATION OF MULTIPLE PRIVACY PROTECTION METHODS

In this step, we determined five privacy protection methods (M1, M2, M3, M4, and M5) with different levels of privacy protection strength in mHealth apps.

#### STEP 2: RECRUITING AND SCREENING OF STUDY PARTICIPANTS

A study advertisement was posted on a website to recruit study participants. Every potential study participant was screened using the Institutional Review Board (IRB) approved selection criteria (University of Pittsburgh IRB protocol ID: PRO18020101). A group of eligible study participants was invited to participate in the study. The study was performed in a locked conference room. Only the investigators (LZ and BP) and one study participant were in the room during each study session.

#### STEP 3. SIGNING OF THE CONSENT FORM

Before the study commenced with a participant, they were given the opportunity to read and sign the IRB approved consent form. Study participation was completely voluntary, and the participants could leave the study at any time.

#### STEP 4. INTRODUCTION OF PARTICIPANTS TO THE STUDY

At the beginning of the study, the investigators explained the purpose of the study, the procedure to be followed, and what data would be collected in the study.

#### STEP 5. DEMONSTRATION OF THE FIVE PRIVACY PROTECTION METHODS

After the study introduction, the investigators demonstrated the five privacy protection methods (M1-M5) used in several mHealth apps.

#### STEP 6. FILLING OUT OF THE QUESTIONNAIRE

The study participants were then asked to fill out a questionnaire that elicited demographic information, their perceived privacy protection level (PPPL) for the five presented privacy protection methods, their preference rating for each method, and the methods they typically used to protect their local privacy. The questionnaire was administered on a 10-inch iPad via the web-based Qualtrics system.

#### STEP 7. INTERVIEW

A few interview questions were asked after these study participants finished the questionnaire to collect more detailed information about the rationale behind their preference ratings and obtain general comments and suggestions on the privacy protection methods used in mHealth apps.

#### STEP 8. DATA ANALYSIS

The collected data was summarized and analyzed to draw conclusions. Further details of each step in the study are provided in the following sections.

### FIVE PRIVACY PROTECTION METHODS

The following paragraphs describe five different privacy protection methods with varying privacy protection strength. The first two are widely used in many mHealth apps and briefly mentioned in the Introduction section. The third is also currently available in some existing multi-module mHealth apps, such as iMHere 1.0 (Parmanto et al., 2013). The last two methods have been newly introduced into the iMHere 2.0 system (Bendixen, et al., 2017; Setiawan et al., 2019) and are not currently used in other mHealth apps.

Method 1 (M1): mHealth apps are named after the name of the target disorder. This is very convenient for marketing purposes as many potential app users may be able to easily find these apps using a keyword search in major app stores. However, M1 has the weakest privacy protection strength (no protection at all) since it is very easy for people around the app user to determine the nature of the app based on the app name.

Method 2 (M2): mHealth apps have a more generic name but are designed for a *single purpose.* M2 is stronger than M1 in terms of privacy protection since one cannot easily determine the nature of the app by its name, but it may not be sufficient for some users or to protect highly sensitive health information since a quick search on the internet can determine the purpose of the apps.

To provide stronger local privacy protection, we proposed and implemented three additional privacy protection methods in iMHere 1.0 and iMHere 2.0 (Bendixen et al., 2017; Parmanto, et al., 2015; Parmanto et al., 2013; Setiawan et al., 2019). Common to these three new methods is that the app is not used to manage a single disease but to manage various personal health tasks in general via different *modules* in the mHealth app. Therefore, other people around the app user cannot determine the user's purpose for installing and using the app simply from the existence of the app on a mobile device or the name of the app. Because of this, the privacy protection strength of these three new methods is stronger than that of M2.

Method 3 (M3): The mHealth app has a generic name (e.g., iMHere) and there are *multiple modules* in the app for various health care purposes such as medication management, skin care, mood assessment, personal health record management, exercise and nutrition tracking, and goal setting. Users can use any one or combination of these modules. All modules are shown in the dashboard of the app.

Method 4 (M4): This method is similar to M3, but with the additional feature that app users can easily hide or unhide the modules using the settings page of the app. This makes M4 stronger than M3 in terms of privacy protection strength.

Method 5 (M5): This method is similar to M4, but module changes cannot be performed using the settings page of the app; instead they can only be done on the secure Web portal associated with the app. App users need to go through user authentication on the Web portal before they can perform module selection. Once the change is made on the Web portal, it is synchronized to the app. M5 has one more layer of privacy protection (i.e., user authentication), making it stronger than M4.

In M3 – M5, data generated by app users in the modules are always stored on a remote secure server. Hiding a module will not impact user-entered data, but simply make the module not visible or accessible on the dashboard of the app.

Please note that, for the purpose of this project, we address only local privacy protection methods. This is independent of any examination of data security and remote privacy in those mHealth apps.

### STUDY PARTICIPANT RECRUITMENT AND SCREENING

After the study protocol was approved by the IRB office at the University of Pittsburgh, we recruited study participants with the following selection criteria: native English speaker, high school or higher education, age between 18 and 65, capable of communicating with others orally and in writing, has mild or moderate depression, and has local privacy concern when using mHealth apps.

Study participants were recruited through an advertisement posted on the Pitt + Me website at the University of Pittsburgh. Potential participants could indicate their interest in this study by clicking on the link of the study on the website. All potential study participants were required to fill out a screening questionnaire via the Web-based Qualtrics system. Study participants were screened according to the aforementioned selection criteria. Study invitations were sent via email to each eligible participant.

During the screening, the severity level of depression was measured using the 9-item Patient Health Questionnaire (PHQ-9) (Kroenke et al., 2001), where a score of 0-4 means no depression, 5-9 means mild depression, 10-14 means moderate depression, 15-19 means moderately severe depression, and 20-27 means severe depression. In this study, only those with a PHQ-9 score between 5 and 14 were screened further for other selection criteria.

We chose people with mild or moderate depression for three reasons. First, these people may not have very obvious symptoms of depression, making it more likely that they would desire to hide this information from others. Second, we did not have a psychiatric professional onsite to handle emergency situations for people with *severe* depression. Third, it did not make sense to ask people without depression to tell us their preferred privacy protection method when using depression-related apps.

We chose people with local privacy concerns when using mHealth apps for one reason. That is, if people don't have this type of privacy concern, they *don't perceive the need* for local privacy protection, and therefore, it does not make sense to ask them their preferences with regard to local privacy protection methods. If they had any preferences, those would be related to other characteristics of mHealth apps, such as the usability, accessibility, and functionality of the apps, which are not the focus of this study.

### DATA COLLECTED DURING THE STUDY

On the questionnaire, the first set of questions (Q1.1-Q1.8) collected demographic information: age, gender, race, education, marital status, employment status, and experience with using smart devices. These questions were followed by a question (Q2) about the privacy protection methods the study participant had used in the past.

- Q2. To prevent others around you from finding out that you have mental health issues through mHealth apps you use, what steps (actions) have you taken to protect your privacy?

The next set of five questions (Q3.1-Q3.5) asked about participants' PPPL for the five methods presented in the study. A description of each privacy protection method was presented followed by a question to elicit participants' PPPL for that method:

- Q3.1. In your opinion, what is the privacy protection level of this method on a scale of 0 - 10 where 0 means no protection at all and 10 means the strongest protection possible?

These five questions were used to determine whether the study participants had a proper understanding of the privacy protection strength of the five methods. In terms of PPPL, the expected output overall should be M5 > M4 > M3 > M2 > M1 since the privacy protection strength is determined by the methods themselves. If the results for PPPL were not in this order, it meant the participants did not fully understand these five methods and their privacy protection strength. In this case, their ratings and preferences provided in the latter part of the study would be problematic.

The third set of five questions (Q4.1-Q4.5) elicited study participants' preference ratings for the five methods. Similar to the previous question set, each privacy protection method description was followed by a request to rate it on a scale of 1-5, least favorable to most favorable:

- Q4.1. Please rate this privacy protection method on a scale of 1 – 5 where 1 means least favorable and 5 means most favorable.

These five questions were used to determine study participants' preferences for each privacy protection method. These ratings were not independent. Each participant had to indicate one *unique* rating (1-5) for each method. Therefore, Q4.1-Q4.5 is essentially one question.

During the interview, the study participants were asked the following two questions and their follow-up questions for clarification purpose: (1) *Please explain the reason for your ratings.* Typically, the follow-up questions of this question were: Why do you prefer this particular method? Why do you think this method is not good? (2) *Do you have any suggestions or comments on privacy protection methods in mHealth apps?* The follow-up questions of this one were: Why do you think this new method is better? Are you willing to enter a password every time you want to use the module or make changes on modules? Notes were taken on study participants' answers, comments, and suggestions and these notes were summarized and arranged into themes.

### MHEALTH APPS USED IN THE STUDY

**Figure 1:**
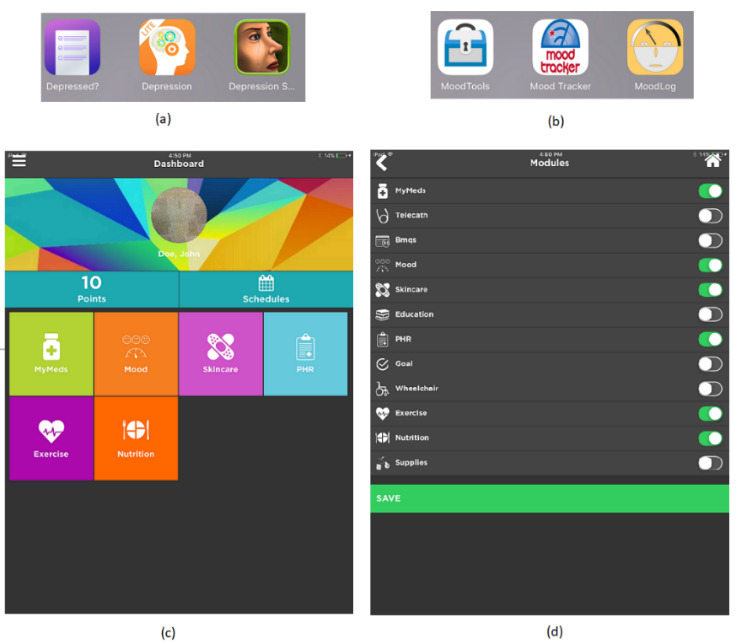
Screenshots of iMHere 2.0 Used in the Study

*Note.* (a) Icons of apps used for M1 demonstration. (b) Icons of apps used for M2 demonstration. (c) A screenshot of iMHere 2.0 dashboard. Six modules (MyMeds, Mood, Skinacre, PHR, Exercise, and Nutrition) are shown. (d) A screenshot of the in-app module selection page in settings of iMHere 2.0. Users can turn on or off each individual module on this page.

Multiple mHealth apps were used in this study to demonstrate the five privacy protection methods (M1-M5). The apps *Depression, Depressed*, and *Depression Symptoms* were used for the M1 demonstration; *MindTools, Mood Tracker*, and *MoodLog* were used for the M2 demonstration. iMHere 2.0 was used for the M3 and M4 demonstration (Setiawan et al., 2019); and iMHere 2.0 and its corresponding Web portal were used for the M5 demonstration. iMHere 2.0 is an app with multiple modules, such as MyMeds for medication management, Mood for mood assessment, Skincare for minor skin problem reporting, PHR for personal health record management, and Exercise and Nutrition for physical activity and nutrition tracking. Figure 1 shows the screenshots of the iMHere 2.0 app.

The focus of the app demonstration was on the local privacy protection methods used by the apps, not on their usability or functionality. We reminded study participants of this focus multiple times during the study (i.e., during the study introduction, method demonstration, and interview). This was to ensure that the preferences study participants expressed were not determined by the quality of the apps but by their privacy protection approach. In fact, the apps used in this study are all excellent mHealth apps. For instance, MoodTools has been rated highly by the Anxiety and Depression Association of America.

### DATA ANALYSIS

Descriptive statistics were calculated for all items on the questionnaire. Statistical significance was determined by p<.05. The normality of the data was evaluated using the Shapiro-Wilk test. A non-parametric test (the Kruskal-Wallis test) was used to determine the statistical significance of the differences among the five privacy protection methods in terms of PPPL. A chi-square test was performed to determine the association between the participants' preference rating and the strength of privacy protection methods. Statistical analyses were conducted using IBM SPSS, version 24.

A power analysis was performed to determine the required sample size. For the mean and mean rank comparisons in this study with a hypothesized difference of 2 and maximum variance of 8, to reach a 95% confidence level and 80% power, the recommended sample size was 32 (Wang & Chow, 2007).

## RESULTS

### DEMOGRAPHICS

In total, 294 people expressed an interest in this study via the Pitt + Me website, 235 of which filled out the screening questionnaire. All 235 potential participants agreed to provide their information to the research team for screening purposes. They all reported being native English speakers and being able to speak English fluently. They were all aged between 18 and 65 years old. They all had received at least a high school level education.

Of these 235 potential participants, 45 (19.1%) were eligible for this study and received a study invitation. Forty-two (42, 93.3%) of these eligible persons accepted the invitation, and 40 (88.9%) attended the study. The major reason for non-eligibility for this study was not having local privacy concerns (160, 68.1%). Among those eliminated who did have local privacy concerns (75, 31.9%), 30 either did not have depression or had moderately severe or severe depression and therefore were not eligible.

The 40 participants' average age was 34.6 (SD=12.13); their average PHQ-9 score was 10.5 (SD=2.75), and average years of using a smart phone or tablet were 7.6 (SD=1.97). The study participants' occupations were highly diverse, and included jobs such as administrative assistant, administrative coordinator, application development manager, chef, computer technician, data analyst, editor, homemaker, human resource manager, librarian, medical records technician, mover, nanny, personal trainer, research assistant, retired person, scientist, software trainer, student, and teacher. A summary of further demographic characteristics is shown in Table 1.

**Table 1:** Demographic Information of the Study Participants (N=40)

	n	%
**Gender**		
Male	18	45.0
Female	22	55.0
**Age**		
18-28	13	32.5
29-50	22	55.0
51-65	5	12.5
**Race**		
Black	4	10.0
White	33	82.5
Other (1 Asian and 2 Hispanic)	3	7.5
**Severity of Depression**		
Mild	18	45.0
Moderate	22	55.0
**Education**		
Up to Associate's degree	14	35.0
Bachelor's degree	16	40.0
Master's or doctoral degree	7	17.5
Professional degree	3	7.5
**Marital Status**		
Single	24	60.0
Married or long-term committed relationship	13	32.5
Divorced or separated	3	7.5
**Employment**		
Employed	29	72.5
Not Employed	5	12.5
Retired or Disabled	6	15.0

### PRIVACY PROTECTION METHODS USED BY STUDY PARTICIPANTS

In Q2 of the questionnaire, study participants were asked to indicate the privacy protection methods they already used. They could choose one or multiple options or give their own answer. The most frequently selected approach was removing browsing history. The next was only using home devices to perform searches. A quarter of participants chose to install and then uninstall apps. Several indicated that they very strictly limited the access to their mobile devices. In other words, if the mHealth apps they used could not provide the desired privacy protection, users took actions and used various methods on their own to protect their privacy. The details are provided in Table 2.

**Table 2:** Privacy Protection Methods used by Study Participants (N=40)

Options	n	%
Delete Internet browser history after your searches	27	67.5
Only use home devices to search for health-related information	21	52.5
Install and uninstall health-related mobile apps	10	25.0
Other (e.g., limit access to my device very strictly)	5	12.5

During the interview, some study participants also mentioned other privacy protection methods they used on their mobile devices. For instance, they arranged their mHealth apps into folders and named them with a common name such as “lifestyle” or placed these folders on pages other than the first page of the app list.

### PARTICIPANTS' UNDERSTANDING OF AND PREFERENCE RATINGS FOR THE FIVE METHODS

Q3.1 – Q3.5 were used to determine study participants' PPPL for the five privacy protection methods (M1-M5) while Q4.1 – Q4.5 were used to collect study participants' preference ratings for the five methods. Table 3 shows the descriptive statistics of answers to these ten questions. From the values, it is clear that M5 > M4 > M3 > M2 > M1 in terms of both PPPL (M1_P – M5_P) and preference rating (M1_R – M5_R), which indicates that study participants had a correct understanding of the privacy protection strength of the five methods and had a higher preference for stronger privacy protection methods, respectively, even though the stronger methods required completing more steps. Further assessment is necessary to determine whether the differences among different methods were statistically significant.

**Table 3:** Perceived Privacy Protection Level and Preference Ratings for the Five Privacy Protection Methods (N=40)

Methods	PPPL (0 - no protection, 10 - strongest protection)			Methods	Preference Rating: 1 - least favorable, 5 - most favorable (n, %)				
	Mean	SD			1	2	3	4	5
M1_P	3.63	2.844		M1_R	35, **87.5%**	4, 10.0%	1, 2.5%	0, 0	0, 0
M2_P	4.75	2.109		M2_R	2, 5.0%	29, **72.5%**	6, 15.0%	3, 7.5%	0, 0
M3_P	5.70	2.564		M3_R	2, 5.0%	4, 10.0%	27, **67.5%**	2, 5.0%	5, 12.5%
M4_P	7.47	1.485		M4_R	0, 0	1, 2.5%	3, 7.5%	23, **57.5%**	13, 32.5%
M5_P	8.27	1.664		M5_R	1, 2.5%	2, 5.0%	3, 7.5%	12, 30.0%	22, **55.0%**

To perform further analysis, the normality of PPPL data was evaluated using the Shapiro-Wilk test. The results indicate that M2_P (p=0.64) and M4_P (p=0.07) were normally distributed. The data for all of the others (M1_P, M3_P, M5_P) were not normally distributed (p<0.05), indicating that when comparing the results for different methods, non-parametric tests would be more appropriate.

To determine whether the differences for the five methods in terms of PPPL were statistically significant, the non-parametric Kruskal-Wallis test for multiple group comparison was performed. The Krusakl-Wallis H test showed that there was a statistically significant difference in PPPL between the different privacy protection methods. The Kruskal-Wallis H score is 78.016, p < 0.001, with a mean rank score of 55.86 for M1, 70.36 for M2, 93.66 for M3, 132.00 for M4, and 150.61 for M5.

A chi-square test was performed to determine whether the participant indicated preference rating was associated with their perceived privacy protection strength. An association between the preference rating and the strength of privacy protection (M5 > M4 > M3 > M2 > M1) was observed, with χ^2^(16) = 332.500, p < 0.001. The result in Table 3 shows that these participants gave higher preference ratings to stronger privacy protection methods.

### RATIONALES FOR THE PREFERENCE RATINGS

During the interview, the study participants explained the reasons for the preference ratings they assigned to the five privacy protection methods. The two major reasons expressed for preferring M4 and M5 were (1) that users could control the display of those modules either in the app or via a Web portal, and (2) that these two methods have stronger privacy protection. The following are the comments from people who preferred stronger privacy protection methods. Please note, the participant IDs were assigned to all potential study participants and they were not updated after some potential study participants were not eligible for the study. Therefore, some labels are bigger than 40.

- *I believe the ability to hide the module is the most preferred for me. I do not think the website ones would be convenient for me. However, I do think the hiding is a feature I need in some way or form. Whether it be via website log in or just hidden in some way. That's a feature I need.* [participant 17]
- *Most secure was rated as most favorable.* [participant 44]
- *Modules with explicit naming give away too much information. Commonly named apps also have the same issue. An app with multiple modules that can be made inaccessible in the setting is good but using a website seems the most secure.* [participant 68]
- *It gives me some privacy with little effort.* [participant 78]
- *Discretion makes it more favorable.* [participant 87]
- *I feel like being able to hide modules gives me a sense of control. I feel more secure sharing data when I don't have to be as concerned that someone may access it when I put my phone down.* [participant 88]
- *I like the ability to control and change my app.* [participant 158]
- *Greater privacy is preferable.* [participant 174]
- *App with multiple modules is simple but still provides sufficient protection.* [participant 201]

The major reason for preferring M2 or M3 over M4 or M5 is that there are more steps in M4 and M5, and some study participants believed that M2 or M3 were good enough for their situation. The following are comments from study participants who assigned a lower preference rating to M4 and M5 and higher rating to M2 or M3.

- *I like the idea of the mood tracker being built into another app, but I am typically the only one who uses my phone and don't think someone will take the time to look up the app I use. While the website-controlled toggling is nice for certain situations, I am not in that situation.* [participant 6]
- *I'd rank the last 3 with same rank if possible. I see the multiple module app as an improvement over the other two, but do not see much difference in the multiple module app options.* [participant 125]
- *Too many options or too many steps may dissuade me although it is a nice option to have.* [participant 126]

### COMMENTS AND SUGGESTIONS ON PRIVACY PROTECTION METHODS

During the interview, study participants were asked to provide comments and suggestions on privacy protection methods. One frequently mentioned approach was to have user authentication before accessing the modules in a mHealth app instead of making module selections on a Web portal. In other words, an alternate version of M5. Following is the rationale for this suggestion from study participants.

- *App with website access may be an unnecessary feature for some people which adds to complexity of privacy situation making it less likely to agree to use that app if you do not have significant information you need to protect.* [participant 201]

For the alternate version of M5, some study participants suggested adding a password before the module change in the settings and some recommended a password or fingerprint for modules with sensitive information, all inside the app, instead of using a Web portal.

- *Maybe entering a password before getting to settings.* [participant 6]
- *I think it would be cool to add a password option on the modules individually to avoid having to hide/unhide modules consistently. It would be my ideal version of this app.* [participant 17]
- *Add a login for sensitive apps or data.* [participant 98]
- *I like a one-step authentication to get into an app, such as fingerprint.* [participant 125]

## DISCUSSION

### PRINCIPAL FINDINGS

mHealth apps can be very helpful in supporting health self-management and intervention delivery if people use these apps in their own health care. However, some people have privacy concerns about mHealth apps and so may choose *not* to use mHealth apps (Atienza et al., 2015; Proudfoot et al., 2010). The general goal of this study was to examine solutions for local privacy protection issues so that people would be more willing to use mHealth apps in their health care. The following is a summary of this study's unique contributions.

First, this study differentiated *local* and *remote* privacy violations since the solutions to each may be different. There are already many methods to prevent remote privacy violations, such as data encryption, access control, user authentication, and user education. Therefore, this study focused specifically on identifying user-preferred approaches for preventing local privacy violations when people use mHealth apps.

Second, it is known that different people have different levels of privacy concerns about their health information and that they may desire different types of privacy protection methods (Zhou et al., 2019); therefore, five methods (M1 – M5) with different privacy protection strengths were presented to study participants so that they could choose the one that was most suitable for them. It is also known that people consider usability of apps when they choose security and privacy features (Smith et al., 2017). Hence, the usability of security and privacy features may affect their preference for privacy protection methods.

Third, a mixed-methods approach (i.e., demonstration, questionnaire, and interview) was used in this study to determine study participants' understanding of the five privacy protection methods, their preferred methods, and the reasons behind their preferences. The results from this study approach should be more reliable than a Web-based questionnaire alone.

Fourth, two user-preferred privacy protection methods for mHealth apps were identified in this study. In these two preferred methods, there are two common components: (a) the mHealth app is *multi-purpose,* and different health-related tasks can be performed via different modules in the app; (b) users can *hide/unhide* modules of the mHealth app. The difference between these two methods is the *location* of the module selection: one is in the app (M4) and the other is on a Web portal (M5). Study participants offered an alternate option for M5, that is, performing user authentication before accessing the setting page for module selection in the app. Our results indicated that app users prefer to use apps with multiple modules designed for different purposes. Other people around the users then cannot determine the users' purpose for installing the app on their mobile device. The results also indicated that it would be even better if the users could customize the modules demonstrated in the dashboard of the app as it would provide another layer of privacy protection.

These two user-preferred privacy protection methods may be used to solve the dilemma that many people are currently facing. That is, they want to use mHealth apps to discover more information about their health problems and identify resources to keep themselves healthy; at the same time, they do not want others around them to know that they have those health problems (Goldenberg et al., 2014; Zhang et al., 2019). This is especially true for people who have health problems *without symptoms noticeable to others around them but that may be associated with stigma*, such as mild or moderate mental health issues and HIV infection (Goedel et al., 2017; Goldenberg et al., 2014, 2015). More specifically, our solution to this dilemma is that the desired mental health or HIV prevention/treatment information may be ***implemented as individual modules in a more generic and multi-purpose mHealth app***, such as iMHere 2.0 (Setiawan et al., 2019). When users need to use the mental health modules or HIV related modules, they can choose these modules in the app or on a corresponding Web portal. These modules would be shown in the dashboard of the app and users can access all the features offered by these modules. After they finish using these modules, users can hide these modules in the app or on the Web portal while their data are stored in an encrypted format on a secure server. Other people around these users would not know if they have used these modules. This approach would satisfy the privacy protection requirement for HIV prevention apps described in a study done by Goedel et al. (2017): “*…, these apps should be designed to be discrete in nature, protect privacy, and not necessarily appear overtly to be related to HIV prevention. Should another individual, for example, see an app explicitly for HIV prevention, it could expose an individual to HIV-related stigma (if others assume they are HIV-positive) and discourage them from using these types of apps.*”

This study was partly motivated by the so-called *privacy paradox*, that some people express their privacy concerns but do not take actions to protect their privacy. For instance, according to a recent survey conducted by the Pew Research Center (2019), 81% of Americans own smartphones, and they have used their smartphones to access highly sensitive health and financial information. While many expressed their security and privacy concerns (Zhou et al., 2019; Zhou, et al., 2018), 28% do not even use the simplest screen lock on their smartphone (Olmstead & Smith, 2017). One possible reason is the inconvenience of using the privacy protection measure in practice. This motivated us to investigate which *privacy protection methods users prefer* in mHealth apps, which are typically the ones that app users identify after they balance the need for privacy protection and the usability of the app.

The finding of this study also indicated that people with local privacy concerns can identify the strong privacy protection methods (M4 and M5 in this study) if they are available. They can balance the usability of the app and their privacy protection needs.

What we (app developers and researchers) need to do is to familiarize more people with these privacy protection methods and encourage other app developers to adopt user preferred privacy protection methods in their apps, which may encourage more people to use mHealth apps that deal with highly sensitive information.

The alternate approach suggested by study participants for M5, the addition of a user authentication method in M4 to access module settings, has both advantages and disadvantages. The advantage is that with this approach, all changes on the modules could be performed in the mHealth app; users would not need to access a separate Web portal to make the desired changes on modules. The specific implementation of the user authentication would determine the disadvantages of this alternate approach. If user authentication were required for individual modules, it would become tedious to enter passwords for access to each of them, especially for modules without highly sensitive information such as records about physical activity and nutrition. If user authentication is only required on modules with sensitive information, *this protection itself could violate local privacy*. That is, others would know the user had used the module (e.g., depression, HIV infection, hepatitis) if it required authentication. Even if user authentication was required only before the module selection page instead of individual modules, others would still know the user is hiding something, even though they do not know the specific content. In other words, requiring user authentication can provide protection on the *content* of the information stored in the app, but *it cannot prevent local privacy violation*. In addition, if there was no corresponding Web portal, the user would not be able to remotely turn off all the modules if the mobile device is lost or stolen.

This study contributes to the limited literature on local privacy protection in mHealth apps. To our knowledge, this is the first study to identify user preferred local privacy protection methods among multiple ones with varying privacy protection strength.

### COMPARISON WITH PRIOR STUDIES

As mentioned in the previous section, we differentiated local and remote privacy violations and specifically identified mHealth app user preferred privacy protection methods for local privacy protection in this study. In previous studies, local and remote privacy violations were not differentiated. Authors in previous studies simply asked their study participants whether they had privacy concerns. There were proposed privacy protection methods in general, but there was no method specifically designed for local privacy protection. Two frequently mentioned privacy protection methods in previous studies were password protection and encryption (Goldenberg et al., 2015; Mitchell et al., 2016), but these are not sufficient for local privacy protection. After all, the existence of a single-purpose app for one disease or password protection for an app may be sufficient to trigger a local privacy violation.

### LIMITATIONS

The sample size of this study (N=40) was not large, but it was sufficient (≥ 32) for mean and mean rank comparison among the five privacy protection methods. In this study, study participants needed to see the demonstration of the five privacy protection approaches before they could make informed decisions. The in-person interview sessions made it feasible for us to understand the reasons behind the study participants' preference ratings. Therefore, although the sample size is not very big, the results from this study are reliable.

As mentioned in the Methods section, we reminded the participants of the study focus multiple times during the study. However, it was inevitable that some study participants might still choose their preferred privacy protection method based on the usability and functionality of the demonstrated apps. For instance, a few participants mentioned that they liked M3-M5 because the iMHere 2.0 app could finish many different health-related tasks in one mHealth app, even though they reported that that was not the only reason for them to have a stronger preference for M3-M5. This is a limitation of using different mHealth apps to demonstrate the five privacy protection methods. It might be better if we had used the same app for the demonstration of all five methods, even though we do not believe this approach would have significantly changed the results. After all, during the app demonstration for M1 and M2, the major focus was the app name, and the first page of these apps, not the detailed functionality of the apps. The major difference between these apps and iMHere 2.0 is the single purpose app vs. module-based multiple purpose app, and this is part of the difference we wanted to have in this study. Our study indicated that many users preferred module-based multi-purpose apps, which are both rich in functionality and provide better privacy protection. These two aspects of this type of app are not separable in terms of this type of local privacy protection methods (M3-M5), and these three methods are all demonstrated in iMHere 2.0.

The alternate approach suggested by study participants was to add a password in M3 or M4 for protecting modules with sensitive information. We believed this approach would not be better than M3 or M4 itself. The reason is that the password is used to protect the confidentiality of the content, while the local privacy violation may occur because of the existence of the module. However, this alternate approach was not explicitly evaluated in this study. Therefore, we could not provide any quantitative results about it. In the future, a different implementation of this approach can be added into the iMHere 2.0 app, and a similar study can be performed to determine whether users have significantly stronger preference to this approach, and which specific implementation for authentication is the most favorable.

## CONCLUSIONS

In this study, user preferred privacy protection methods were identified. mHealth apps with multiple modules for different purposes can provide stronger privacy protection than single-purpose apps, and users preferred to use privacy protection methods with a module selection option available for hiding or unhiding modules with sensitive information.

It is desired that more mobile app developers choose to use a module-based approach in their apps, which can provide more flexible and user-preferred privacy protection. Such module-based apps may encourage more users to adopt mHealth apps and better manage their own health.
